# The phytohormone forchlorfenuron decreases viability and proliferation of malignant mesothelioma cells *in vitro* and *in vivo*

**DOI:** 10.18632/oncotarget.27341

**Published:** 2019-12-10

**Authors:** Walter Blum, Thomas Henzi, László Pecze, Kim-Long Diep, Christian G. Bochet, Beat Schwaller

**Affiliations:** ^1^Section of Medicine, University of Fribourg, CH-1700 Fribourg, Switzerland; ^2^Department of Chemistry, University of Fribourg, CH-1700 Fribourg, Switzerland

**Keywords:** malignant mesothelioma, septin cytoskeleton, septin 7, forchlorfenuron, FCF

## Abstract

Malignant mesothelioma (MM) is one of the most aggressive cancer types with a patient’s life expectancy of typically less than one year upon diagnosis. The urgency of finding novel therapeutic approaches to treat mesothelioma is evident. Here we tested the effect of the plant-growth regulator forchlorfenuron (FCF), an inhibitor of septin function(s) in mammalian cells, on the viability and proliferation of MM cell lines, as well as other tumor cell lines derived from lung, prostate, colon, ovary, cervix and breast. Exposure to FCF strongly inhibited proliferation of human and mouse (most efficiently epithelioid) MM cells and all other tumor cells in a concentration-dependent manner and led to cell cycle arrest and cell death. The role of septin 7 (*SEPT7*), a presumably essential target of FCF in MM cells was confirmed by an shRNA strategy. FCF was robustly inhibiting tumor cell growth *in vitro* at low micromolar (IC_50_: ≈20-60µM) concentrations and more promisingly also *in vivo*. Initial experiments with FCF analogous revealed the importance of FCF’s chloride group for efficient cell growth inhibition. FCF’s rather low systemic toxicity might warrant for an extended search for other related and possibly more potent FCF analogues to target MM and putatively other septin-dependent tumors.

## INTRODUCTION

Malignant mesothelioma (MM) is an extremely aggressive tumor arising from the pleural, peritoneal and pericardial mesothelial cell layer, in most cases after asbestos exposure [1]. An estimated 107,000 – 112,000 people worldwide die each year from mesothelioma, lung cancer and asbestosis [2]. Conventional first line treatment for MM consists of chemotherapy (cisplatin and pemetrexed) and more recently also immunotherapy. Chemotherapy is now frequently accompanied by radiotherapy and/or surgery (for review, see [3–5]) indicating that multimodality treatment appears as the most promising MM treatment strategy. Patient survival is extended on average by only about 1 year by the various treatments and significant improvements in increasing patient survival require the development of new treatment strategies and identification of novel molecules with an appropriate and efficient pharmacological profile, even if recent multimodality trials in early-stage pleural MM show promising results [6]. Recently, the regulation of calretinin, the most sensitive and selective marker for MM [7, 8] by septin 7, was demonstrated [9]. Overexpression of septin 7 in MM cells decreases calretinin expression levels by septin’s binding to the calretinin (*CALB2*) promoter, thus acting as a negative transcriptional regulator. Similarly, calretinin overexpression reduces septin 7 levels indicative of a possibly reciprocal, antagonistic regulation; yet mechanistic details on the latter, i.e. calretinin decreasing septin 7 expression are currently unknown. Septins are considered as the fourth component of the cytoskeleton besides actin, microtubules and intermediate filaments. The human genome encodes 13 septin genes classified into 4 groups based on sequence similarity [10]. Some septin family members (30-65 kDa) are expressed ubiquitously, while others show tissue-specific expression patterns. They are highly conserved, GTP-binding and membrane-interacting proteins and belong to the Ras-like GTPase superclass of phosphate-binding loop NTPases. All septins form higher-order structures such as filaments, bundles, scaffolding structures or rings [10]. The different septins are implicated in various cellular processes such as actin dynamics, cytoskeleton organization, cytokinesis and membrane dynamics; often they serve as scaffolds for protein recruitment and as diffusion barriers for subcellular compartmentalization. Members of all 4 subgroups interact strongly with other septin family members; moreover, various septin-interacting proteins have been described forming the septin interactome [11]. The presence of septin 7 is indispensable for cytokinesis in fibroblasts; deficiency causes incomplete cytokinesis and constitutive multinucleation. Constitutive deletion of septin 7 leads to early embryonic lethality in mice [12]. To date, studies on the role of septin 7 in cancer are still rare. Septin 7 expression in various glioma cell lines was found to be reduced compared to normal human glia cells, while overexpression suppressed glioma cell migration. Septin 7 functions as a tumor suppressor also in thyroid carcinoma; expression levels in carcinoma samples are lower compared to benign thyroid nodules [13]. However, higher expression levels of septins 2/7 in breast cancer [14] and hepatocellular carcinomas (septin 7) hint towards a putative role also as oncogenes in those cancer types [15].

The synthetic urea derivative forchlorfenuron (FCF) is a phytohormone with cytokinin activity and is used worldwide as a plant growth regulator that increases fruit size. Since its development in the 1980s, FCF has been widely used in grapes, kiwifruits, apples, pears and watermelons [16, 17]. In 2004, Iwase et al. [18] reported that FCF treatment caused defects in cytokinesis and deformation of septin filaments in budding yeast; the effects are rapid and reversible upon removal of FCF. FCF alters septin assembly *in vitro* without affecting actin or tubulin polymerization. In HeLa and MDCK cells, both of epithelial origin, septin organization and dynamics are modified by stabilizing septin filaments resulting in cell morphology changes, mitotic defects and decreased cell migration [19]. Moreover, FCF induces septin polymerization and stabilizes extended septin polymers reversibly [20]. Cell detachment triggers redistribution of septins to the plasma membrane and formation of microtentacles. This process is inhibited by FCF in breast, lung, prostate and pancreas cancer cells *in vitro* indicating that septins play an essential role in the metastatic behavior of tumor cells [21].

The low toxicity level of FCF, which was thoroughly investigated by the United States Environmental Protection Agency (EPA) makes thus FCF a promising candidate for putative therapeutic applications in cancers with elevated septin levels and/or increased septin function. Here we tested the effect of FCF on cells of mesothelial origin, with a focus on MM cells. In all cases FCF efficiently blocked proliferation of MM cells *in vitro* and pilot experiments with the murine MM cell line AB12 revealed that FCF might also be applied for MM treatment *in vivo*.

## RESULTS

### FCF exposure decreases proliferation of cells of mesothelial origin

**Figure 1:**
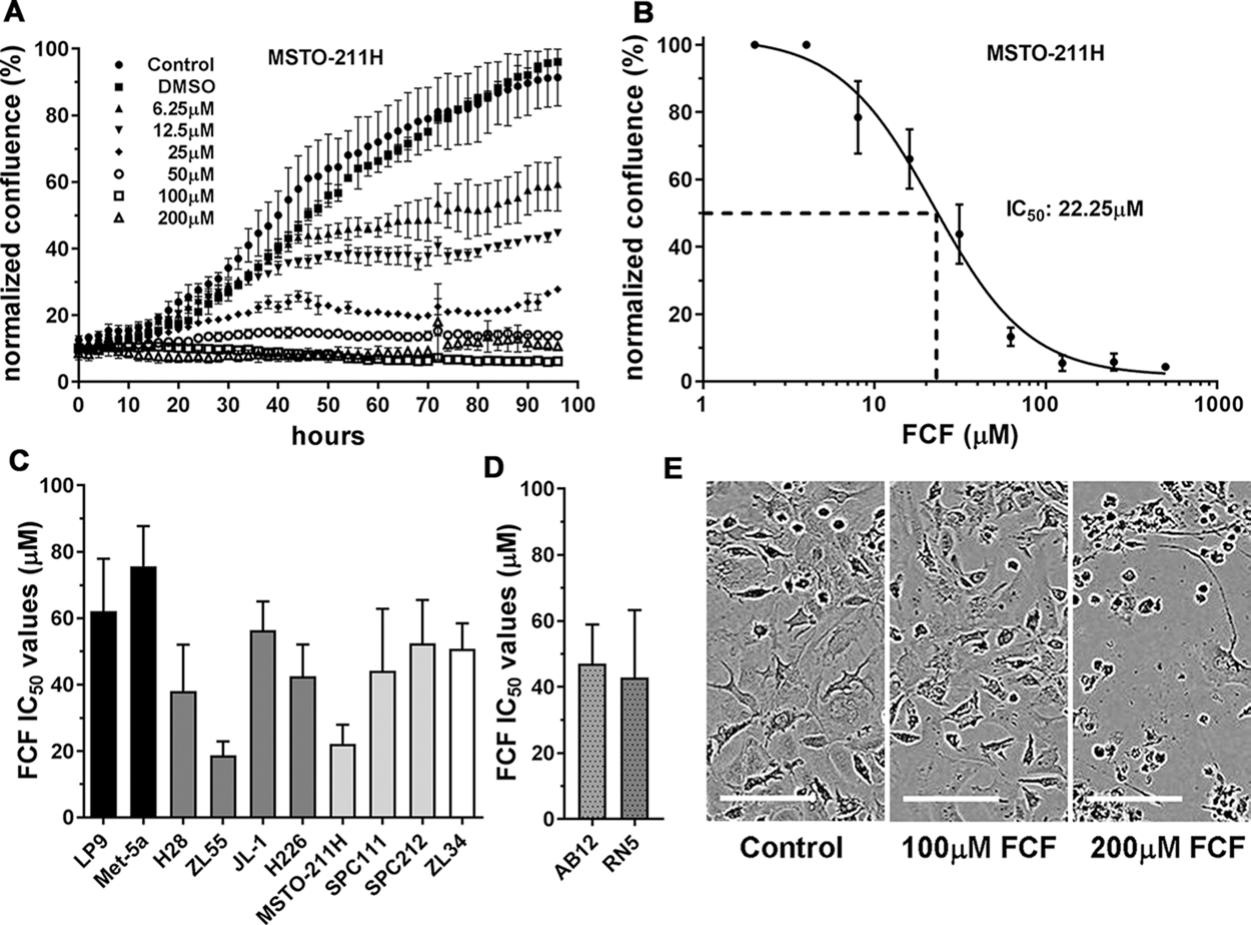
Proliferation-inhibiting effect of FCF in cells of mesothelial origin. **(A)** Human MSTO-211H cells were exposed to FCF in a concentration range from 6.25 µM to 200 µM and monitored for a period of 96 h. Growth curves from a representative experiment are shown. The symbols show the average value from 6 wells ± SD. At least 3 experiments were carried out in identical experimental conditions. **(B)** Determination of IC_50_ of FCF in MSTO-211H cells. The concentration of FCF required for 50% inhibition of proliferation was calculated as 22 µM. **(C)** IC_50_ values of FCF determined in human immortalized mesothelial cell lines (black bars) and human MM cell lines derived from epithelioid (dark grey), biphasic (light grey) and sarcomatoid (white) MM. **(D)** IC_50_ values of FCF determined in mouse MM cell lines from BALB/c (AB12) and C57Bl/6J (RN5) mice. **(E)** Toxicity testing in a confluent layer of immortalized iMeso-WT1 mesothelial cells exposed to 100 and 200 µM FCF. At 200 µM FCF, a strong cytotoxic effect is observed, while 100 µM was tolerated without apparent signs of toxicity. Scale bar: 100 µm.

Human MSTO-211H cells derive from a biphasic MM, and when grown *in vitro*, mostly consist of cells with an epithelioid morphology, with few spindle-shaped cells. Cells were grown *in vitro* and exposed to FCF at concentrations ranging from 6.25 µM to 200 µM; cell proliferation was monitored using the Incucyte live-cell imaging system (Figure 1A). Since FCF was initially dissolved in DMSO, cells grown in the presence of the same final DMSO concentration (≤0.5%) served as a negative control; MSTO-211H growth curves were essentially identical in the presence or absence of 0.5% DMSO. An inhibitory effect on MSTO-211H cell proliferation was observed already at the lowest concentration applied (6.25 µM); starting from approximately 40 h after FCF treatment, the slopes of the curves leveled off reaching a plateau evident at concentrations ≥12.5 µM. At concentrations ≥50 µM proliferation had almost totally stopped. The resulting IC_50_ value for FCF was calculated to be approximately 22 µM (Figure 1B). These initial results prompted us to test the effect of FCF in a series of cells of mesothelial origin, mostly human MM cell lines; IC_50_ values ranged from 19 µM (ZL55) to 56 µM (JL-1) (Figure 1C). The effects of FCF on cell proliferation (real-time growth curves) are additionally shown for murine RN5 MM cells (supplementary Figure 1). Besides real-time growth curves, FACS analyses with FCF-treated MM cells (50 µM, 24 h) were carried out. In all tested cell lines (human MSTO-211H and ZL55, mouse AB12) the increase of the G2/M peak was indicative of a cell cycle block at G2/M (supplementary Figure 2). In support of an inhibition of cell proliferation, the fraction of Ki67-positive cells was strongly diminished in FCF-treated ZL55 and AB12 cells (supplementary Figure 3).

For comparison of effects in MM cells *vs.* non-transformed mesothelial cells we included the two immortalized non-tumorigenic cell lines Met-5A and LP9/TERT-1. IC_50_ values were higher in Met-5A and LP9/TERT-1 cells (76 and 62 µM, respectively) than in MM cell lines, indicative of a lower sensitivity of non-transformed mesothelial cells to the growth-inhibiting/cytotoxic effects of FCF. On average, epithelioid MM-derived cells (H28, ZL55, JL-1, H226) showed a slightly higher sensitivity to FCF than most MM cells derived from biphasic MM (MSTO-211H, SPC111, SPC212) or the sarcomatoid MM-derived ZL34 cells. This is in line with the observation that patients diagnosed with a sarcomatoid MM are in general more resistant to the first-line chemotherapy consisting of cisplatin and pemetrexed treatment [8].

The sensitivity to FCF was also tested in the two murine MM lines AB12 and RN5, both used in further *in vitro* and *in vivo* experiments (see below); IC_50_ values in both cell lines were similar to human MM cell lines: 47 µM and 43 µM, respectively. Since local chemotherapy is clinically relevant for the treatment of MM we tested the toxicity of FCF on a confluent layer of immortalized mouse mesothelial iMeso-WT1 cells [22], presumed to mimic the situation prevailing in the peritoneal cavity and similar to a clinical setting (Figure 1E). iMeso-WT1 cells tolerated up to 100 µM FCF without evidence of cytotoxicity, while at the higher FCF concentration of 200 μM massive cell death evidenced by detached cells, cell shrinkage and membrane blebbing was observed. A similar outcome was also observed in ZL55 cells exposed to 80 µM FCF (supplementary Movies M1 and M2). Since expression of septins is not unique to cells of mesothelial origin, the effect of FCF exposure was tested in a collection of cell lines derived from solid tumors of the lung, prostate, colon, ovary, cervix and breast. All tumor cell lines from our cell collection were strongly affected by FCF in a dose-dependent manner; exposure to 100 μM FCF resulted in a >80% inhibition of proliferation in all cell lines (supplementary Figure 4). In particular, most colon-derived tumor cells (WiDr, HT29, Co115 [23]) showed high sensitivity to FCF already at 50 µM.

### Spindle-shaped cells from biphasic MM are less sensitive towards FCF-induced proliferation arrest/cytotoxicity compared to cells with epithelioid morphology

**Figure 2:**
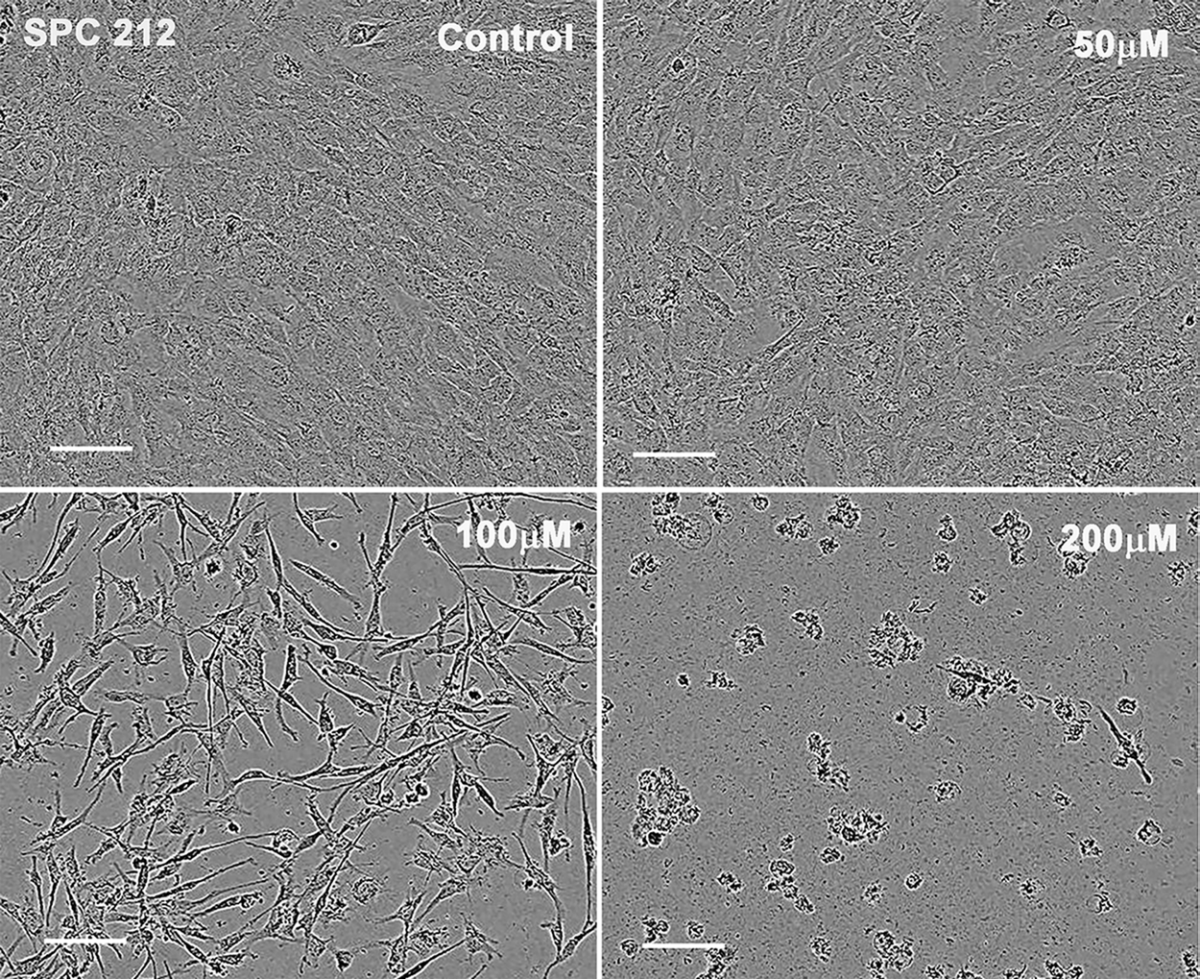
Stronger cytotoxic effect of FCF on the population of SPC212 cells with epithelioid morphology than cells with sarcomatoid morphology. SPC212 cells, representing one of the most FCF-resistant MM cell lines, were exposed to 50, 100 and 200 µM of FCF for 96 h. At 50 µM FCF cells were barely affected, while at 100 µM FCF preferentially cells with sarcomatoid morphology survived the treatment; most epithelioid cells had disappeared. At 200 µM almost all cells of both morphology types died. Scale bar: 100 µm.

To confirm our observation that human sarcomatoid MM cells were more resistant to the FCF proliferation-inhibiting/cytotoxicity effects than epithelioid MM cells (Figure 1C), we exposed SPC212 cells to various FCF concentrations ranging from 50 – 200 µM (Figure 2). SPC212 cells are derived from a biphasic MM, and when cultured as a cell monolayer in a culture flask *in vitro*, a mixture of cells with epithelioid-like and fibroblast-like (spindle-like) cells are present (Figure 2). Treatment with 50 μM FCF marginally increased the proportion of spindle-like cells without apparent signs of cytotoxicity. Exposure to 100 μM FCF revealed that the morphology of surviving cells was mostly elongated (spindle-like), while cells with epithelioid appearance disappeared. In order to exclude that the change in cell morphology (lengthening/stretching) of surviving SPC212 cells was merely the effect of a lower cell density resulting from cell death of a fraction of FCF-treated cells, control SPC212 cells were seeded at various lower densities to reach a comparable confluence as observed in FCF-treated (75 µM and 100 µM) cells. The morphology of untreated SPC212 cells in low-density cultures was noticeably different from that of FCF-treated cells: there was still a considerable number of SPC212 cells with an epithelioid morphology, despite a large amount of unoccupied space (supplementary Figure 5), indicating that low cell density is not a crucial factor contributing to the mostly elongated cell morphology. Of note, the elongations were extremely thin resulting in a needle-shaped morphology essentially absent also in low-density SPC212 cultures. However, higher FCF resistance of spindle-like cells might not be the only cause; it cannot be excluded that upon FCF treatment some epithelioid SPC212 cells changed their morphology to spindle-like cells. Finally, FCF (200 μM) induced massive cell death in both types of cells resulting in very low overall cell survival after 4 days of treatment. To further investigate the effect of FCF on septin organization, SPC212 cells seeded at a relatively low density were immunostained for septin 7. Septin filaments (resembling actin-based stress fibers) were most clearly seen in epithelioid and to a lesser extent in spindloid SPC212 cells (supplementary Figure 6). In FCF-treated (50 µM & 75 µM) SPC212 cells, septin 7 distribution was clearly different: in some cells septin 7 was accumulated in small puncta aggregates in the cytosol and along the plasma membrane, while in others filaments appeared thinner and in (likely dying) cells characterized by nuclear blebbing, septin 7 staining was rather homogeneous (blurry) with some stronger stained puncta (supplementary Figure 6). Yet in some SPC212 cells, staining was very similar as in control cells. Of note, septin 7 staining intensity was not noticeably weaker in FCF-treated cells confirming that FCF mostly affects septin filament structure/organization, not septin 7 expression.

### Specific downregulation of septin 7 via lentivirus-mediated Sept7 shRNA leads to similar changes in proliferation and induction of cell death as FCF treatment

**Figure 3:**
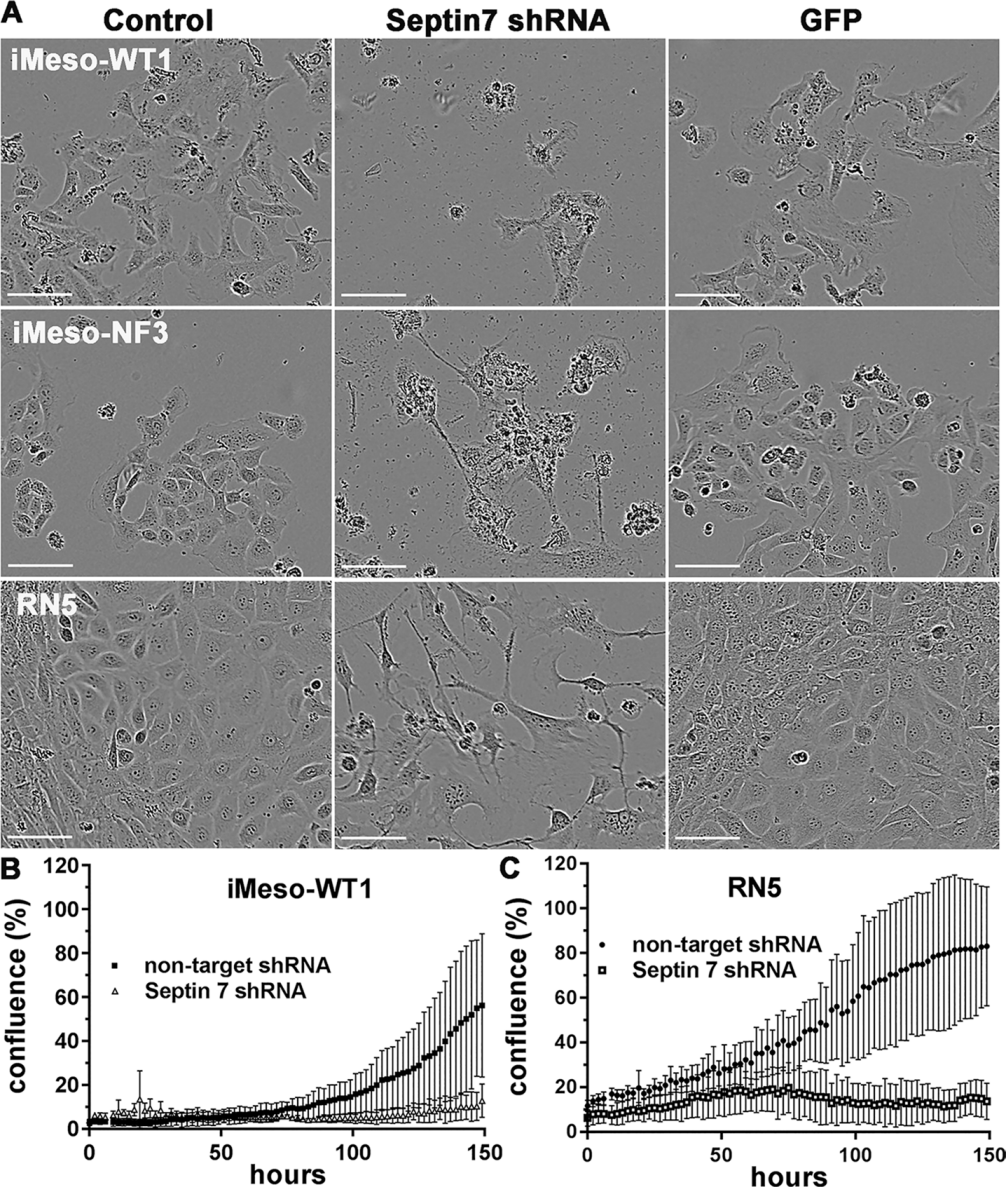
Septin 7 downregulation decreases viability of both murine immortalized mesothelial cells and MM cells. **(A)** Untreated murine immortalized mesothelial cell lines iMeso-WT1 and iMeso-NF3 and RN5 MM cells (left panels) consist of cells with epithelioid and sarcomatoid morphology. Effect of shRNA-mediated downregulation of septin 7 (middle panels). In all 3 cell lines, reduced septin 7 expression resulted in massive cell death and survival of cells with mostly sarcomatoid morphology as observed at 100 h post LV infection. LV infection with pLV-GFP leading to the expression of GFP served as a negative control (right panels). No morphological changes were observed in all 3 GFP-expressing cell lines. Scale bar: 75 µm. **(B)** Real-time growth curves determined in iMeso-WT1 cells (control (non-target) cells or LV-shSEPT7 infected and grown for 150 h) showed the same effect, i.e. complete block of proliferation in absence of septin 7. **(C)** Identical results are observed in RN5 MM cells.

In view of our final aim to study the inhibiting effects of FCF on MM growth in a syngeneic mouse model *in vivo*, the next series of experiments were carried out with the mouse MM cell line RN5, established in asbestos-exposed C57Bl/6J mice. As a control we used previously described immortalized mouse mesothelial cells either generated from primary mesothelial cells of a WT mouse (iMeso-WT1) or from a mouse heterozygous for the tumor suppressor merlin (*Nf2*), named iMeso-NF3, both lines also derived from C57Bl/6J mice [22]. First we evaluated, whether the FCF-mediated effects observed in human MM cells were caused by the inhibition of septin 7 function(s) and not by other potential off-target effects [24]. For this, we directly investigated the effect of selective septin 7 downregulation by an shRNA approach. We transduced RN5, iMeso-WT1 and iMeso-NF3 cells with a previously validated, commercially available and potent shRNA against septin 7 (Figure 3A). Down-regulation of septin 7 caused rapid cytostatic and cytotoxic effects evidenced 72 h post-transduction in all 3 cell lines investigated. Cells were inept to carry out cytokinesis and in most cases, underwent cell death following prolonged cytokinesis. The effects of septin 7 downregulation were even more pronounced in the two immortalized, mesothelial cell-derived lines iMeso-WT1 and iMeso-NF3 indicating that septin 7 downregulation also has a strong effect in untransformed (normal) mesothelial cells. As a negative control for lentivirus-mediated transgenesis, transduction with the lentivirus pLV-GFP resulting in the expression of *GFP* mRNA and subsequently GFP, had no obvious cytotoxic effect in all tested cell lines. In addition, real-time cell growth curves of murine iMeso-WT cells (Figure 3B) and RN5 cells (Figure 3C) were acquired with the Incucyte Live-cell imaging system. Control cells of both lines, treated with a non-targeted (scramble) shRNA, showed normal proliferation during the entire duration of the experiment (150 h; compare to growth curve of untreated MSTO-211H cell shown in Figure 1A). On the contrary, shRNA-mediated downregulation of septin 7 strongly inhibited growth of both cell lines. The growth-inhibiting effect was detectable approximately 35 h after LV infection and the cytotoxic effect was discernable 75 h after the beginning of the treatment.

### Side effects observed in FCF-treated mice *in vivo*

**Figure 4:**
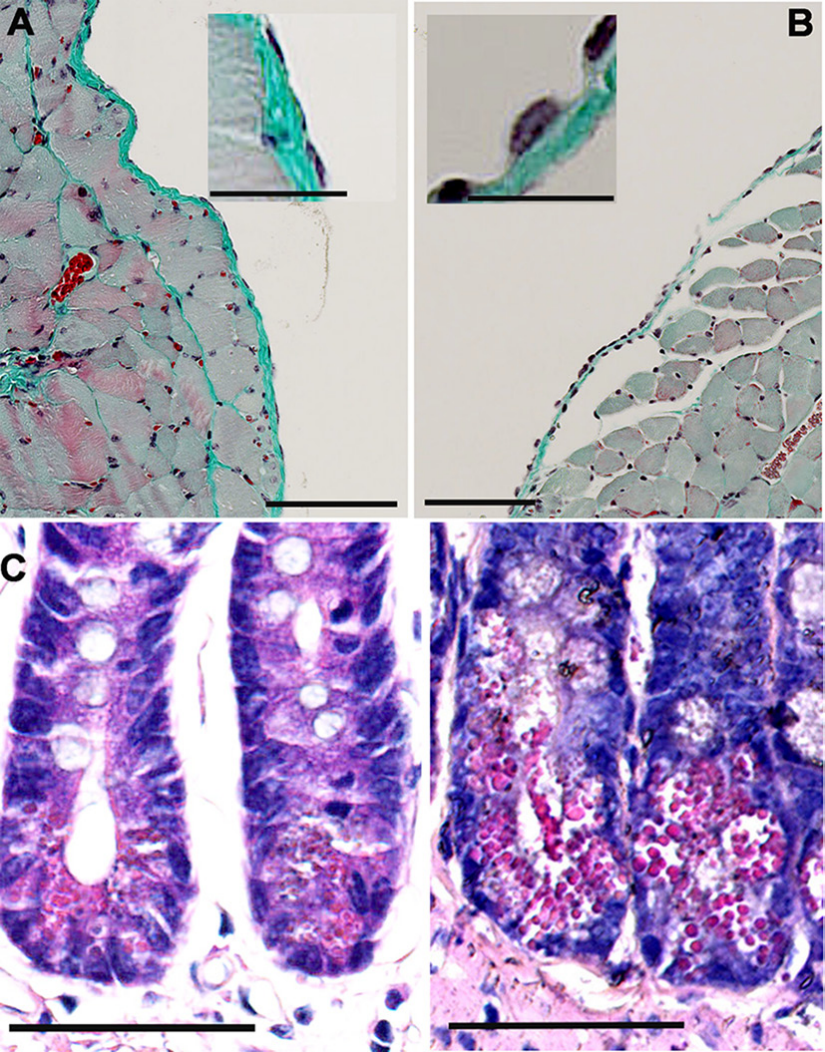
Histopathological analyses of FCF-treated mice. **(A)** Untreated control mouse shows a normal mesothelium consisting of a monolayer of mesothelial cells on top of (loose) connective tissue (green) covering the peritoneal side of the diaphragm. The typical flat (squamous) morphology of mesothelial cells is seen in the inset. Scale bars: 100 µm in the large image, 25 µm in the inset; Goldner staining. **(B)** Following intraperitoneal FCF injection (9 days post-injection), a thickening of the mesothelium, mostly the result of a morphological change from a flat to cuboid morphology of mesothelial cells is observed, typical for reactive mesothelial cells (inset). Scale bars: 100 µm in the large image, 25 µm in the inset. **(C)** Longitudinal sections (Azan staining) of intestine of a control mouse (left) and of an FCF-treated mouse for 9 days (right). Abnormal dying cells with Paneth cell-like morphology characterized by a high number of apical red-stained granules are more numerous in tissue from FCF-treated mice. Scale bar: 100 µm.

FCF has a relatively short half-life, shows low systemic *in vivo* toxicity and is thus a U.S. government-approved fertilizer used in fruit horticulture. However, essentially nothing is known about FCF effects, if injected into the peritoneal cavity. Mice subjected to intraperitoneal FCF treatment (25 mg/ml dissolved in PG; 75 µl (1.875 mg) per mouse) generally showed, as for most cytostatics, symptoms including partial alopecia, heavy constipation and few mice were found in hypothermia, the latter were subsequently euthanized. To analyze the side effects of FCF on the tissues present in the peritoneal cavity, we performed histological analyses of various abdominal samples (Figure 4). The diaphragm –like the abdominal cavity walls– as well as the internal organs is covered by a mesothelium; the single layer of mesothelial cells form the barrier between the tissue and the peritoneal cavity. The single squamous layer of mesothelial cells in a control mouse is seen as the lining of the diaphragm (Figure 4A). In FCF-exposed mice (Figure 4B) the mesothelial layer had a more cuboidal appearance, characteristic for reactive mesothelial cells, often resulting from an inflammation or injury of the mesothelium. In addition, we investigated the wall of the intestinal tube. While we didn’t observe striking effects in the tunica serosa (except the reactive mesothelial cells) and the tunica muscularis (not shown), the rapidly regenerating intestinal epithelium was clearly affected by the FCF treatment. At the base of the Lieberkuhn crypts, which preferentially contain proliferating stem cells and Paneth cells, a higher fraction of cells had flatter (compressed) nuclei and the abundance of red-stained secretory granules was higher. The number of such “Paneth-like” cells per crypt and at times the content of granules per cell were increased. How these FCF-induced morphological alterations might be linked to the observed constipation in some of the FCF-exposed mice warrants further investigation. Also other drugs used in chemotherapeutic regimens that kill rapidly proliferating cells, e.g. vinca alkaloids (vincristine, vinblastine and vinorelbine) acting on the tubulin cytoskeleton show –as a side effect– constipation in patients [25] similar as FCF (here shown in mice) that affects the septin cytoskeleton.

### FCF treatment decreases the growth of AB12 MM cells in a syngeneic orthotopic mouse model *in vivo*

**Figure 5:**
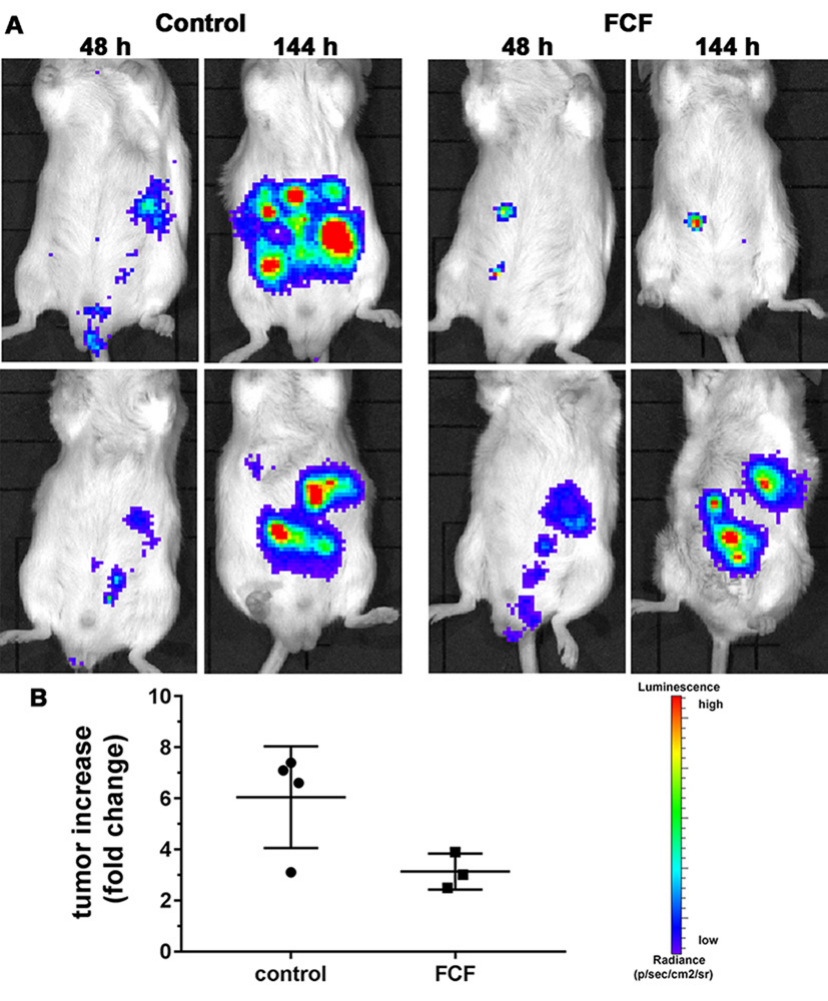
Tumor growth of AB12 MM cells *in vivo*. **(A)** BALB/c mice were injected with 1x10^5^ syngeneic AB12-LV-hRluc MM cells. FCF (or saline) was administered intraperitoneally 48 h later. Tumor growth was monitored by *in vivo* bioluminescence imaging at 48 h (before FCF treatment) and at 144 h after AB12 cell injection (96 h FCF treatment or control). BLI signal intensity changes from blue (low) to red (high). Images from 2 mice per group are shown. **(B)** Quantification of the fold increase in BLI signal intensity between 48 h and 144 h post-injection (4 days of treatment). The time point 48 h served as reference point (normalization) in all mice (control mice: n=4, FCF-treated mice: n=3).

In order to test the effect of FCF on MM cell proliferation/tumor growth *in vivo*, we injected 1x10^5^ AB12-pLV-hRluc cells (originating from a BALB/c mouse) intraperitoneally into BALB/c mice. These cells are tumorigenic in syngeneic (BALB/c) immunocompetent mice [26]. In a pilot experiment, 48 h after injection of the AB12 cells and after the first BLI measurement (basal value for each mouse), FCF (25 mg/ml in PG, 100 µl (2.5 mg) per mouse) or 100 µl saline was administered via intraperitoneal injection. Representative results of *in vivo* bioluminescent optical imaging are presented in Figure 5A. The increase in the bioluminescence signal from 48 to 144 h representing tumor growth was evident in untreated control mice (left panel). In FCF-treated mice, the increase in BLI signal intensity was lower indicative of reduced/impaired tumor growth (right panel). The quantification of the bioluminescence data (Figure 5B) yielded a fold change of 6.05 for control mice (n=4) *vs.* 3.13 (p=0.063, t-test) for FCF-treated mice (n=3).

### Structural analysis of FCF derivatives reveals the chloride group as essential for effectiveness of inhibition of MM cell proliferation

**Figure 6:**
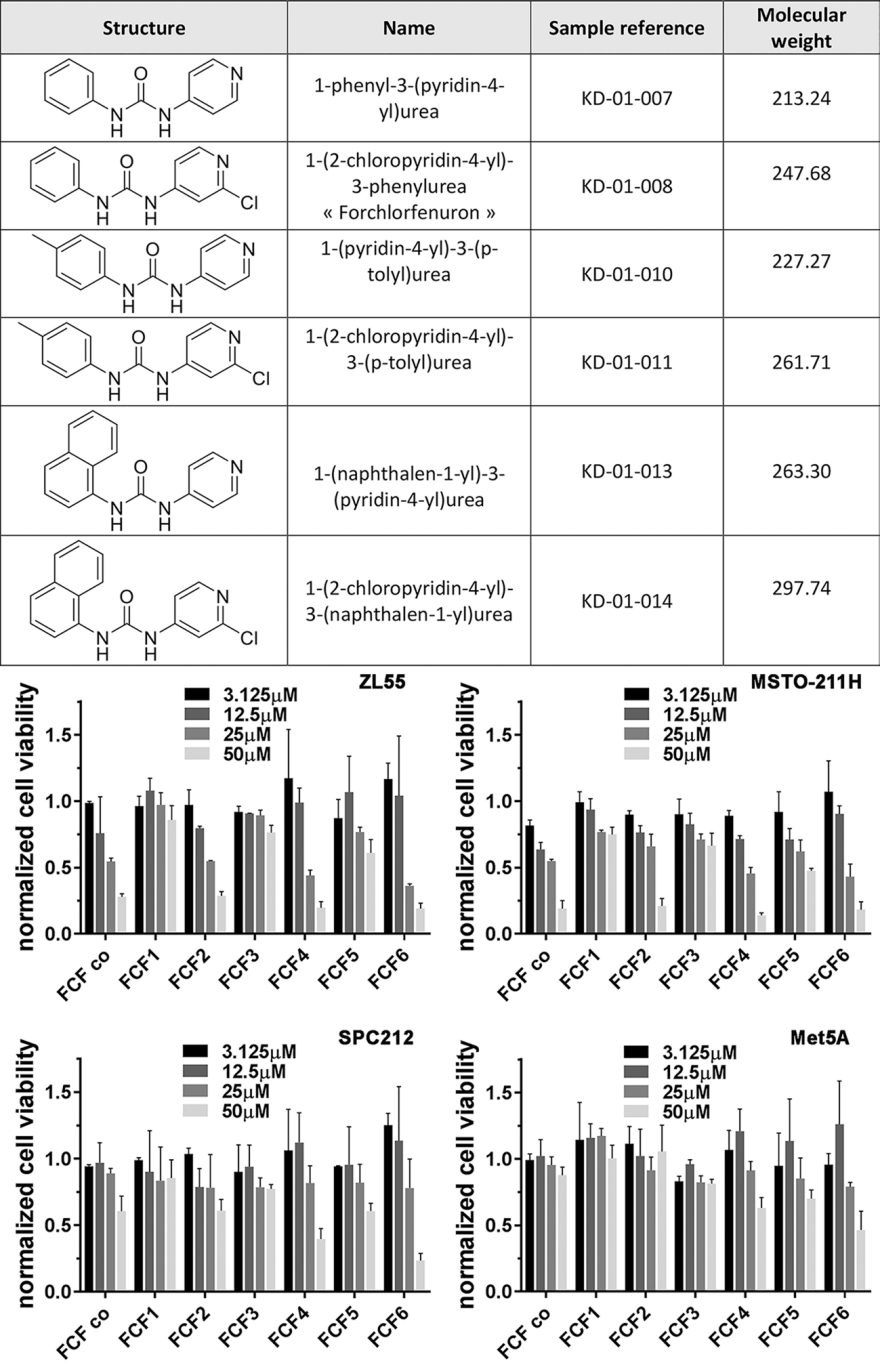
MM growth-inhibiting effects of FCF derivatives *in vitro*. Top) Description and characteristics of different diarylureas used for determining growth inhibition/viability. **Bottom)** MM cell lines ZL55, MSTO-211H and SPC212 and Met-5A mesothelial cells were exposed to the substances listed at various concentrations for 96 h. Normalized (to untreated control cells) MTT signal intensities are shown for the 4 cell lines tested (n = 3 experiments, 3 wells/FCF derivatives FCF1-6). FCF_co and FCF-2 are chemically identical, but from different sources. Values are mean ± SD.

Experiments were carried out to identify structural moieties in FCF required for the efficient blocking of MM cell proliferation. For this, besides the commercially available FCF, 6 diarylureas comprising FCF and 5 other substances closely related to FCF were synthetized (Figure 6, top). The main reasoning for using two batches of FCF was to exclude that the way of synthesis and/or purification might have affected the results. The effect of the two batches of FCF, the commercially obtained one (FCF_co) and the newly synthesized FCF2 were almost identical in all 4 cell lines tested (Figure 6, bottom). Three different groups of substances were tested. In the first one (substance 1), the chloride in the pyridine ring of FCF was omitted. In the second group (± chloride), the phenyl ring was changed to a p-tolyl ring and in the third group (± chloride) the phenyl ring was replaced by a naphtalen-1-yl moiety. The 6 substances (all at concentrations between 3.125 – 50 µM) were tested in the MM cell lines ZL55 (epithelioid), MSTO-211H (biphasic with a relative high content of epithelioid cells) and SPC212 (biphasic with a majority of sarcomatoid cells). In addition the effect of the synthesized substances on the non-transformed mesothelial cell line Met-5A was investigated. The results shown in Figure 6 can be summarized as follows: 1) Only molecules containing the chloride group (substances 2, 4, 6) were efficient in blocking MM proliferation in a dose-dependent manner. The identical substances without a chloride group (1, 3, 5) showed a much weaker (if any) effect on MM cell proliferation. This indicates the importance of the chloride group likely for the high-affinity binding of FCF to septins. The addition of the methyl group to the phenyl ring (substance 4) or even replacing it by a naphtyl group (substance 6) had a very minor effect on proliferation; inhibiting effects were nearly identical as with genuine FCF (substance 2). 2) The growth inhibiting effects of substances 2, 4 and 6 containing the chloride group were much diminished in Met-5A cells demonstrating that MM (transformed) cells show a much higher susceptibility to FCF and analogues than immortalized mesothelial cells. 3) Addition of the bulkier naphtyl group (substance 6) resulted in a stronger proliferation-inhibiting effect also in Met-5A cells, a probable undesired effect, if the drug should allow to distinguishing between transformed MM cells and mesothelial cells.

## DISCUSSION

The need for novel treatment options for some tumor types, including MM, is evident. The results of our study showing the effects of the plant growth regulator FCF on the proliferation and viability of MM cells *in vitro* and *in vivo* are promising. It is well known that septins are implicated in the development of various diseases. Septin 7 is linked to Alzheimer’s disease, schizophrenia, neuropsychiatric erythematosus lupus and different tumors [27]. Nevertheless targeting septins still is a rather neglected research topic. Development of small molecule inhibitors disturbing higher-order assembly of septins would considerably advance our knowledge on the molecular and cellular biology of septins [28].

Exposure of cells of mesothelial origin to FCF had a strong inhibiting effect on proliferation and furthermore decreased viability. The most sensitive cells were epithelioid-type mesothelioma cells, while sarcomatoid cells seemed less impaired. However, septin 7 distribution was clearly affected by FCF also in more spindloid cells such as SPC212. As FCF targets septin filament assembly and functions we downregulated septin 7 by a lentiviral approach using septin 7 shRNA to confirm the septin-dependent effect of FCF. Again, cells of the epithelioid type were more prone to growth inhibition. The experimentally determined IC_50_ value of FCF in MSTO-211H cells was 22.2 μM. This value is comparable to the value given for cisplatin in the dataset found in the Cancer Web portal http://www.cancerrxgene.org of 20.7 μM. Other MM cell lines in the database were in the range of 4.5 – 97 μM. Thus, FCF has a similar *in vitro* cytotoxicity as Cis-Pt, which is used as first line treatment in MM. In mesothelioma cell lines, FCF exposition caused an increase in the proportion of cells in the G2/M phase of the cell cycle pointing to blockage of the terminal step of cell division (supplementary Figure 2). Thus, previously demonstrated calretinin downregulation [9] and septin 7 inhibition by FCF (this study) appear to have the strongest effect on inhibiting MM cell proliferation in cells with high expression levels of the respective proteins (high septin 7 in SPC212 cells, high calretinin and septin 7 in MSTO-211H cells) [9].

In silico, FCF interacts preferentially with the nucleotide-binding pocket of septins involved in GTP-binding and hydrolysis. Hence, septins are stabilized in a conformation that mimics a nucleotide-bound state, preventing further GTP binding and hydrolysis and interfering with the GTP-binding dynamics and turnover of higher-order structures of septins. In more detail, “FCF’s pyridine ring interacts with the Thr-186 residues of both SEPT2 protomers and Gly-241 of protomer A. Notably, the side chain hydroxyl group of Thr-186 (protomer A) is predicted to form a halogen bond with the chlorine atom of FCF’s pyridine ring” [29]. The acceptor capability of organic halogen (Cl, F, Br, I) is still understudied in macromolecules [30], but such halogen interactions are hypothesized to stabilize protein-ligand interactions [31]. Our *in vitro* results in MM cells underpin the importance of this (FCF) chloride-protein interaction, since all FCF analogues without a chloride group had essentially no effect on cell proliferation at the concentrations tested. Septins stabilize ErbB2, an important oncoprotein, in gastric cancer cells. Thus, inhibition of septin oligomerization by FCF causes ubiquitination of ErbB2 at the plasma membrane and subsequent internalization and intracellular degradation [32]. Likewise, disruption of filamentous septin structures by FCF reduces the tumorigenic properties like proliferation, migration and transformation in prostate cancer cells via ubiquitination and degradation of HIF-1α protein, a process normally prevented by filamentous septin structures [33]. Besides, FCF is able to suppress breast cancer cell proliferation and invasion; in particular SEPT2 and SEPT7 seem essential for cell migration and invasion. Given its ubiquitous expression, SEPT7 might be a fundamental molecule, which protects or stabilizes other septin proteins [14].

Our bioluminescent setup in immunocompetent mice demonstrated the *in vivo* applicability of FCF. Growth of tumors induced by injection of tumorigenic AB12 cells was considerably decreased when mice were treated with FCF by intraperitoneal administration. Most MM are localized in the pleural cavity and not, as in our mouse model, intraperitoneal. Administration of chemotherapeutics directly into the pleural cavity is relatively uncommon but it has been shown that it can be conducted safely [34].

The toxicity of FCF is considered to be low, generally limited to decreased body weight and body weight gains. It is classified as not likely to be a human carcinogen (Pesticide Fact Sheet, EPA, 2004). The *in vitro* cytotoxicity of FCF has been evaluated before by Hu *et al.* [19]. The short-term viability of HeLa and MDCK cells (4 h) is not affected even at FCF concentrations of 500 μM. Longer treatments (24 h) are well tolerated up to 62.5 μM FCF. On the other hand, non-negligible off-target effects were reported before [24]: in cultured mammalian cells, mitochondria fragment at FCF concentrations previously assumed to target septins only. In our experiments, intraperitoneal FCF treatment of mice induced as side effects obstipation and alopecia, effects that are often observed during chemotherapies. Histological analysis of the digestive system revealed modifications in the mucosa of the small intestines probably linked to the described obstipation.

FCF is a synthetic urea derivative consisting of a chlorinated pyridine and a phenol ring joined together by a urea group; it can be chemically modified at various positions as demonstrated in this study. A more comprehensive and rigorous investigation on FCF modifications might lead to the development of new analogues with possibly stronger cytostatic or cytotoxic potency in MM cells and less side effects offering additional treatment options for MM and other solid tumors.

## MATERIALS AND METHODS

### Cell culture

Met-5A (SV40-immortalized human mesothelial cells) were obtained from ATCC, cells were grown in Dulbecco’s modified Eagle’s medium/F-12 1:1 plus GlutaMax (Gibco, Basel, Switzerland) supplemented with 10% FCS (fetal calf serum), 100 U/ml penicillin and 100 µg/ml streptomycin (1% PS, Gibco). LP9/TERT-1 cells (TERT-immortalized human mesothelial cells) were obtained from the laboratory of Dr. James Rheinwald (Dana Farber Cancer Research Institute, Boston, MA). The cells were cultured in a medium consisting of 1:1 M199 and MCDB10 supplemented with 15% NCS (newborn calf serum), 5 ng/ml epidermal growth factor (EGF), 0.4 µg/ml hydrocortisone, 2 mM glutamine and 1% PS as described before [35]. The MM cell lines ZL55, SPC111, SPC212 and ZL34 [36] (obtained from the University Hospital, Zurich, Switzerland), JL-1 (from Leibniz-Institute DSMZ) MSTO-211H, H28, H226 (from ATCC), the murine MM cell lines AB12 and RN5 [22] and the immortalized mouse mesothelial cells iMeso-WT1 and iMeso-NF3 [22] were maintained in RPMI1640 (Gibco) supplemented with 10% fetal bovine serum (FBS, Gibco), 100 U/ml penicillin and 100 μg/ml streptomycin (1% PS). WiDr, HT29, CO112, CO115, MCF7, OVCAR, PC3, LNCAP, Du145, A549, HeLa cells were obtained from ATCC. Primary mouse mesothelial cells were isolated and maintained in culture as described in previous studies [37].

### Lentiviral constructs, vector production and lentivirus production

The pLKO.1-shRNA plasmid encoding a validated shRNA targeted to mouse septin 7 (*Sept7*) was purchased from Sigma (Buchs, Switzerland). The Addgene plasmids psPAX2 (plasmid #12260), pMD2.G-VSVG (plasmid #12259) and pLVTHM (plasmid #12247) were a kind gift from Didier Trono (Lausanne, Switzerland). Lentivirus producing an shRNA against septin 7 or an shRNA against GFP were produced and isolated as described previously [35].

### Treatment of cells with FCF

Cells were seeded in 96-well plates (500 cells per well) and grown for 24 h. FCF (CAS 68157-60-8, LabForce AG, Muttenz, Switzerland) was added in a concentration range from 0 to 500 μM and MTT assays were performed 96 h post-treatment to determine the number of viable and/or proliferating cells [9]. Real-time cell proliferation curves and images were acquired with the Incucyte Live-cell imaging system (Essen Bioscience, Ann Arbor, MI) as described before [37].

### *In vivo* bioluminescent optical imaging (BLI) in AB12 cell-injected mice treated with FCF

Murine AB12 MM cells were transduced with lentivirus encoding pLV-hRluc resulting in stable expression of the reporter Renilla Luciferase [38]. BLI was performed in mice injected intraperitoneally with 1x10^5^ AB12-LV-hRluc cells using the IVIS Lumina II *In Vivo* Imaging System (Caliper Life Sciences, Hopkinton, USA). Directly before BLI each mouse received an intraperitoneal injection (1 mg/kg) of luciferase substrate ViviRen™ *In Vivo* Renilla Luciferase Substrate (Cat#P1231; Promega, Dübendorf, Switzerland) following the manufacturer’s instructions. Two time points were chosen for BLI, i.e. 48 h after AB12-LV-hRluc cell injections (before treatment) and 144 h after cell injection (4 days FCF treatment). For the treatment, FCF was dissolved in propylene glycol (PG) to 25 mg/ml and 100 µl (or 100 µl PBS control) was injected intraperitoneally (n=4 per group). For BLI, mice were kept at 37ºC and under 2-3% isoflurane/l/min O_2_ anesthesia during image acquisition and FCF injection. Images were acquired for 5-30 s depending on signal strength. The flux (photons/seconds) in a Region of Interest (ROI) placed over the peritoneal region of the mice was used to quantify BLI signals on the acquired images using the Living Image 4.2 Software. Values of BLI signals are expressed as photons/s/cm^2^/sr.

### Histopathology

Mice were exposed to FCF (i.p. injection; 2.5 mg in 100 µl PG) for 9 days and different organs collected for histopathological analysis, e.g. digestive tract, diaphragm, parietal abdominal wall. Samples were processed as described previously [37]. Briefly, samples were fixed with 4% paraformaldehyde for 48-72 h, dehydrated and embedded in paraffin; then 4 µm sections were cut and subjected to hematoxylin and eosin (H & E) and/or Goldner staining. Representative images were selected from scans of a Hamamatsu whole-slide imaging system (Nanozoomer 2.0-HT).

### Immunofluorescence

SPC212 cells were seeded on glass coverslips in 24-well plates. After fixation for 15 min with 4% paraformaldehyde (37°C), non-specific binding sites were blocked by incubation with TBS containing donkey serum (5%) for 1 h. The rabbit polyclonal anti-septin 7 antibody (Bethyl Laboratories, Montgomery, TX, USA) was diluted 1:2,500 in TBS and coverslips were then incubated overnight at 4°C. After washing, the secondary antibody (Alexa Fluor 488-conjugated donkey anti-rabbit IgG 1:400, Jackson Immunoresearch Laboratories, West Grove, PA, USA) was added for 3 h at room temperature. Nuclei were stained with Hoechst 33342 (1μg/ml, 10 min; Sigma-Aldrich Chemie GmbH, Buchs, Switzerland). Next, the coverslips were mounted with Hydromount solution (National Diagnostics, Atlanta, GA). A Leica TCS SP5 confocal microscope with a 63x glycerol-immersion objective (1.3 NA, Plan APO) was utilized for the image acquirement.

### FACS analysis

MM cells (MSTO-211H, ZL55, AB12) grown in the presence or absence of FCF (50 µM) were trypsinized and centrifuged; cell pellets were washed three times with PBS and resuspended in PBS. FACS analysis was performed on a BD Accuri C6 instrument (BD Biosciences) to quantify the fraction of cells in the different phases of the cell cycle (G1, S, G2/M) using propidium iodide (PI) staining as described before [39]. FACS histograms were analyzed using the FlowJo software (Tree star) and the results are presented as pie charts (supplementary Figure 2). The fraction of Ki-67 positive ZL55 and AB12 cells was also determined by FACS on the same instrument using a Ki-67 antibody (BD Bioscience, Allschwil, Switzerland) according to the manufacturer’s instructions. For quantification of histograms the FlowJo software was used.

### Synthesis and characterization of FCF analogues

Details on the synthesis and characterization of the various diarylureas used in this study are described in the suppl. Material section.

### Statistical analysis

Statistical analyses were performed using StatPlus (AnalystSoft) software and GraphPad Prism (GraphPad Software, San Diego, California, United States).

## SUPPLEMENTARY MATERIALS FIGURES







## Abbreviations

BLI: bioluminescent optical imaging
*CALB2*: gene encoding calretinin
EPA: Environmental Protection Agency
FCF: forchlorfenuron
FCS: fetal calf serum
LV: lentivirus
MM: malignant mesothelioma
NCS: newborn calf serum
ROI: region(s) of interest
*SEPT7*: gene encoding septin 7
