# Supplementary material for: The phytohormone forchlorfenuron decreases viability and proliferation of malignant mesothelioma cells in vitro and in vivo
Source: Oncotarget. 2019 Dec 10;10(65):6944–56. doi: 10.18632/oncotarget.27341 (PMC6916748; doi:10.18632/oncotarget.27341)
Supplement: Supplementary file 1 [file oncotarget-10-6944-s001.pdf]

The phytohormone forchlorfenuron decreases viability and proliferation of malignant mesothelioma cells *in vitro* and *in vivo*

SUPPLEMENTARY MATERIALS

SYNTHESIS AND CHARACTERIZATION OF DIARYLUREAS

GENERAL METHODS

Chemicals were obtained from commercial suppliers (TCI, Acros organics, Sigma-Aldrich, Fluorochem). Dry toluene was obtained from drying columns from the Chemistry Department of the University of Fribourg. Thin layer chromatographies were performed on Merck TLC Silicagel 60 F_{254} or TLC Silica gel 60 RP-18 $F_{254}S$. UV light (254 nm) and/or a potassium permanganate stain were used to visualize the spots on TLCs. NMR measurements were carried out on a Bruker Avance 300 MHz spectrometer (^{1}H: 300 MHz, ^{13}C: 75 MHz) or a Bruker Avance III HD 400 Mhz spectrometer (^{1}H: 400 MHz, ^{13}C: 101 MHz). Chemical shifts are expressed in parts per million using residual solvent protons as internal standards. Coupling constant (*J*) are reported in Hz. Splitting patterns are designated as s (singlet), d (doublet), dd (double doublet), t (triplet), dt (double triplet), q (quartet), br. s. (broad singlet), m (multiplet). Deuterated solvents were obtained from Cambridge Isotope Laboratories. HR-MS measurements were performed on a FT-MS 4.7T Bio Apex II mass spectrometer from Bruker Daltonics. IR spectra were recorded on a Bruker FT-IR Tensor II using a Golden Gate diamond ATR system. Melting point measurements were performed using a Büchi Melting Point B-540 device.

GENERAL PROCEDURE FOR THE SYNTHESIS OR DIARYLUREAS

Ureas were synthesized by reacting aryl-isocyanates with 4-aminopyridines in toluene at 80°C. To a solution of the isocyanate (1.2 eq) in dry toluene the 4-aminopyridine was added (1 eq). The solution was heated at 80°C for 4 to 6 h. If precipitation occurred upon cooling to room temperature, the solution was filtered to obtain the crude product. If not, the solvent was removed under reduced pressure. In both cases, the crude mixture was recrystallized in acetonitrile to obtain the desired diarylurea. Naphtylureas either did not require any recrystallization or were purified by other methods.

1-phenyl-3-(pyridin-4-yl)urea

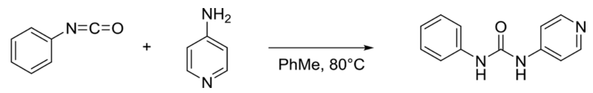

Phenyl isocyanate (0.693 mL, 6.38 mmol, 1.2 eq) and 4-aminopyridine (500 mg, 5.31 mmol, 1 eq) were added to 10 mL of dry toluene in a sealed tube under argon. The reaction mixture was heated at 80°C for 4 h. The solvent was then removed and the solids were recrystallized in acetonitrile to afford 1-phenyl-3-(pyridin-4-yl) (692 mg, 61% yield) as white crystals.

^{1}H NMR (300 MHz, DMSO-d_6) δ = 9.10 (s, 1 H), 8.87 (s, 1 H), 8.36 (d, *J* = 6.1 Hz, 2 H), 7.61 - 7.36 (m, 4 H), 7.36 - 7.20 (m, 2 H), 7.12 - 6.90 (m, 1 H)

^{13}C NMR (75 MHz, DMSO-d_6) δ = 152.1, 150.2, 146.5, 139.1, 128.8, 122.4, 118.5, 112.2

ESI-HRMS *m/z* calcd for $C_{12}H_{11}N_3ONa$ $[M + Na]^+$ 236.0794, found 236.0795

FT-IR (Golden Gate) ν_{max} 3387 (w), 3242 (w), 3185 (w), 3122 (w), 2912 (w), 2851 (w), 2791 (w), 1713 (m), 1621 (w), 1597 (w), 1582 (m), 1556 (m), 1507 (s), 1486 (m), 1443 (m), 1417 (m), 1331 (m), 1317 (m), 1289 (m), 1253 (m), 1190 (s), 1061 (m), 1024 (m), 997 (m), 905 (m), 892 (s), 822 (m), 749 (s), 692 (s), 639 (m) cm^{-1}

mp 160-162°C

1-(2-chloropyridin-4-yl)-3-phenylurea

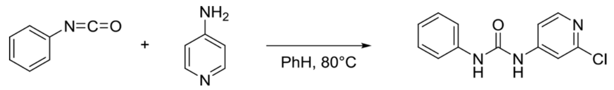

Phenyl isocyanate (0.507 mL, 4.67 mmol, 1.2 eq) and 2-chloro-pyridin-4-amine (500 mg, 3.89 mmol, 1 eq) were added to 10 mL of dry benzene in a sealed tube under argon. The reaction mixture was heated at 80°C for 6 h. After cooling to room temperature, the suspension was filtered and the resulting solids were then recrystallized

in acetonitrile to afford 1-(2-chloropyridin-4-yl)-3-phenylurea (450 mg, 47% yield) as white crystals.

^{1}H NMR (300 MHz, DMSO-d_6) δ = 9.36 (s, 1 H), 8.99 (s, 1 H), 8.19 (d, *J* = 5.7 Hz, 1 H), 7.66 (d, *J* = 1.7 Hz, 1 H), 7.55 - 7.41 (m, 2 H), 7.41 - 7.24 (m, 3 H), 7.14 - 6.96 (m, 1 H)

^{13}C NMR (75 MHz, DMSO-d_6) δ = 152.3, 151.4, 150.5, 149.6, 139.3, 129.3, 123.2, 119.2, 112.4, 111.7

ESI-HRMS *m/z* calcd for $C_{12}H_{11}N_3OClNa$ [M + Na]$^+$ 270.0405, found 270.0401

FT-IR (Golden Gate) ν_{max} 3402 (w), 3246 (w), 3146 (w), 3130 (w), 3029 (w), 2984 (w), 1730 (m), 1583 (s), 1527 (s), 1495 (m), 1472 (m), 1439 (m), 1394 (m), 1304 (m), 1270 (m), 1193 (s), 1127 (m), 1075 (m), 1045 (m), 1025 (m), 989 (m), 934 (m), 876 (m), 833 (s), 790 (m), 748 (m, 715 (m, 687 (m), 636 (m), 607 (m) cm^{-1}

Mp 167-168°C

1-(pyridin-4-yl)-3-(p-tolyl)urea

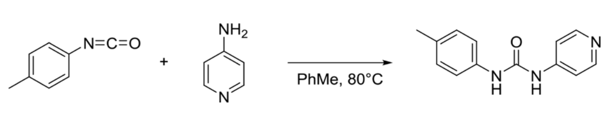

p-Tolyl isocyanate (0.643 mL, 5.10 mmol, 1.2 eq) and 4-aminopyridine (400 mg, 4.25 mmol, 1 eq) were added to 10 mL of dry toluene in a sealed tube under argon. The reaction mixture was heated at 80°C for 5 h. The solvent was removed and the resulting solids were then recrystallized in acetonitrile to obtain 1-(pyridin-4-yl)-3-(p-tolyl)urea (756 mg, 78% yield) as white crystals.

^{1}H NMR (300 MHz, DMSO-d_6) δ = 9.06 (s, 1 H), 8.76 (s, 1 H), 8.44 - 8.28 (m, 2 H), 7.48 - 7.39 (m, 2 H), 7.39 - 7.28 (m, 2 H), 7.20 - 6.99 (m, *J* = 8.3 Hz, 2 H), 2.25 (s, 3 H)

^{13}C NMR (101 MHz, DMSO-d_6) δ = 152.1, 150.1, 146.5, 136.5, 131.3, 129.2, 118.6, 112.2, 20.3

ESI-HRMS *m/z* calcd for $C_{13}H_{13}N_3ONa$ [M + Na]$^+$ 250.0951, found 250.0948

FT-IR (Golden Gate) ν_{max} 3395 (w), 3337 (w), 3277 (w), 3192 (w), 3111 (w), 3028 (w), 2997 (w), 2857 (w), 2801 (w), 1718 (s), 1609 (m), 1582 (m), 1550 (m), 1505 (s), 1489 (s), 1416 (w), 1322 (m), 1289 (m), 1250 (m), 1211 (m), 1188 (s), 1061 (m), 1037 (m), 995 (m), 854 (m), 809 (s), 731 (m), 709 (m), 693 (m), 605 (m) cm^{-1}

Mp 188-189°C

1-(2-chloropyridin-4-yl)-3-(p-tolyl)urea

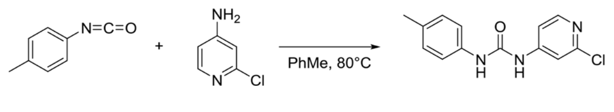

p-Tolyl isocyanate (0.588 mL, 4.67 mmol, 1.2 eq) and 2-chloro-pyridin-4-amine (500 mg, 3.89 mmol, 1 eq) were added to 10 mL of dry toluene in a sealed tube under argon. The reaction mixture was heated at 80°C for 6 h. After cooling to room temperature, the suspension was filtered and the resulting solids were then recrystallized in acetonitrile to obtain 1-(2-chloropyridin-4-yl)-3-(p-tolyl)urea (626 mg, 62% yield) as white crystals.

^{1}H NMR (300 MHz, DMSO-d_6) δ = 9.31 (br. s., 1 H), 8.87 (br. s., 1 H), 8.17 (d, *J* = 5.7 Hz, 1 H), 7.65 (d, *J* = 1.7 Hz, 1 H), 7.44 - 7.22 (m, 3 H), 7.11 (d, *J* = 8.3 Hz, 2 H), 2.25 (s, 3 H)

^{13}C NMR (101 MHz, DMSO-d_6) δ = 151.8, 150.9, 149.9, 149.2, 136.2, 131.6, 129.2, 118.9, 111.8, 111.2, 20.3

ESI-HRMS *m/z* calcd for $C_{13}H_{12}N_3OClNa$ [M + Na]$^+$ 284.0561, found 284.0561

FT-IR (Golden Gate) ν_{max} 3402 (w), 3331 (w), 3264 (w), 3156 (w), 3062 (w), 2989 (w), 2920 (w), 1709 (m), 1579 (s), 1504 (s), 1471 (s), 1390 (m), 1312 (m), 1293 (m), 1267 (m), 1247 (w), 1189 (s), 1123 (m), 1075 (m), 1040 (m), 987 (w), 934 (w), 826 (w), 813 (s), 769 (m), 744 (w), 715 (m), 635 (w), 609 (w) cm^{-1}

Mp 182-183°C

1-(naphthalen-1-yl)-3-(pyridin-4-yl)urea

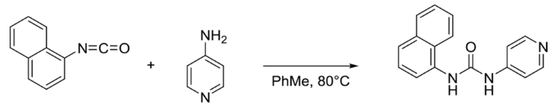

1-Napthyl isocyanate (0.772 mL, 5.10 mmol, 1.2 eq) and 4-aminopyridine (400 mg, 4.25 mmol, 1 eq) were added to 10 mL of dry toluene in a sealed tube under argon. The reaction mixture was heated at 80°C for 5 h. The white precipitate was then filtered, washed with ice cold toluene and placed in a flask. To the flask was then added chloroform (50 mL) and the mixture was heated at reflux for 30 minutes. The mixture was then allowed to cool down to room temperature and the solution was filtered. The filtrate was then evaporated under reduced pressure to obtain 1-(naphthalen-1-yl)-3-(pyridin-4-yl)urea (748 mg, 67% yield) as a white powder.

^{1}H NMR (400 MHz, DMSO-d_6) δ = 9.44 (s, 1 H), 8.93 (s, 1 H), 8.38 (d, *J* = 6.2 Hz, 2 H), 8.10 (d, *J* = 8.4 Hz, 1 H), 7.96 (td, *J* = 1.1, 6.7 Hz, 2 H), 7.69 (d, *J* = 8.3 Hz, 1 H), 7.66 - 7.53 (m, 2 H), 7.53 - 7.43 (m, 3 H)

^{13}C NMR (101 MHz, DMSO-d_6) δ = 152.6, 150.2, 146.5, 133.7, 133.6, 128.4, 126.3, 126.0, 125.9, 125.8, 123.7, 121.4, 118.3, 112.2

ESI-HRMS *m/z* calcd for $C_{16}H_{13}N_3ONa$ [M + Na]$^+$ 286.0951, found 250.0954

FT-IR (Golden Gate) ν_{max} 3270 (w), 3164 (w), 3048 (w), 2988 (w), 1673 (m), 1594 (m), 1529 (s), 1496 (s), 1432 (m), 1418 (m), 1395 (m), 1344 (m), 1331 (m), 1312 (m), 1269 (m), 1250 (w), 1207 (s), 995 (w), 816 (m), 786 (m), 767 (m), 728 (w), 670 (w), 642 (w) cm^{-1}

Mp 225-228°C

1-(2-chloropyridin-4-yl)-3-(naphthalen-1-yl)urea

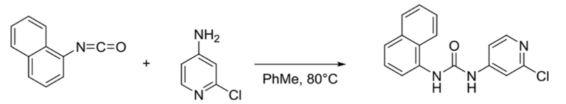

1-Napthyl isocyanate (0.707 mL, 4.67 mmol, 1.2 eq) and 2-chloro-pyridin-4-amine (500 mg, 3.89 mmol, 1 eq) were added to 10 mL of dry toluene in a sealed tube under argon. The reaction mixture was heated at 80°C for 5 h. The white precipitate was then filtered, resulting in 1-(2-chloropyridin-4-yl)-3-(naphthalen-1-yl)urea (720 mg, 62% yield) as a white powder.

^{1}H NMR (400 MHz, DMSO-d_6) δ = 9.68 (br. s., 1 H), 9.03 (br. s., 1 H), 8.20 (d, *J* = 5.7 Hz, 1 H), 8.08 (d, *J* = 8.4 Hz, 1 H), 8.01 - 7.84 (m, 2 H), 7.82 - 7.67 (m, 2 H), 7.67 - 7.45 (m, 3 H), 7.35 (dd, *J* = 1.9, 5.7 Hz, 1 H)

^{13}C NMR (101 MHz, DMSO-d_6) δ = 152.5, 151.0, 150.0, 149.2, 133.7, 133.3, 128.4, 126.6, 126.0, 125.9, 125.7, 124.1, 121.5, 118.9, 111.9, 111.2

ESI-HRMS *m/z* calcd for $C_{13}H_{12}N_3OClNa$ [M + Na]$^+$ 320.0561, found 320.0560

FT-IR (Golden Gate) ν_{max} 3235 (w), 3050 (w), 1670 (m), 1594 (m), 1526 (m), 1432 (m), 1417 (w), 1391 (w), 1344 (m), 1330 (m), 1313 (m), 1269 (w), 1252 (w), 1207 (w), 992 (m), 860 (m), 823 (m), 787 (s), 768 (s), 750 (m), 736 (m), 641 (s), 626 (s), 607 (s) cm^{-1}

Mp 230°C (slow decomposition)

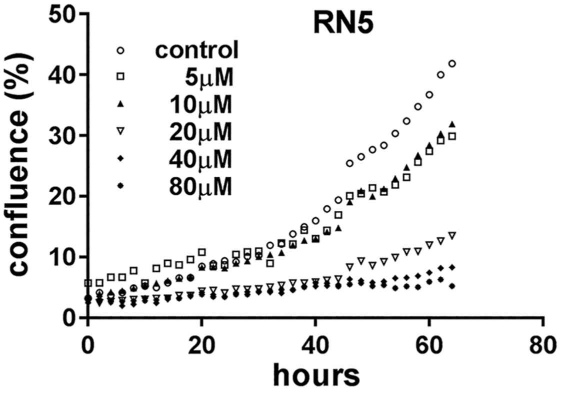

Supplementary Figure 1: Proliferation of murine MM RN5 cells exposed to FCF concentrations ranging from 5 μM – 80 μM was monitored by live-cell imaging.

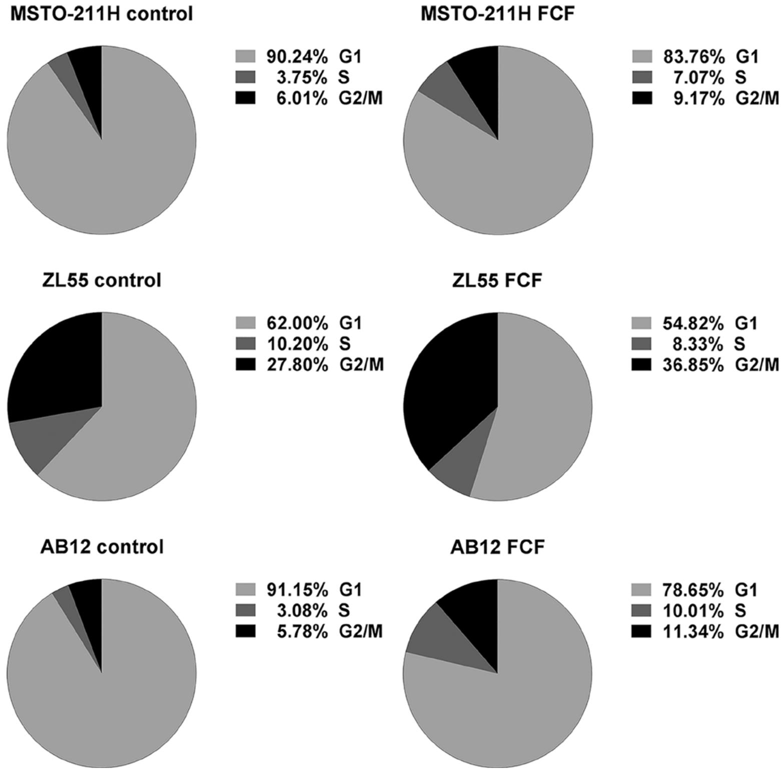

Supplementary Figure 2: MSTO-211H (top), ZL55 (middle) and AB12 (bottom) MM cells were exposed for 24 h to 50 μM FCF. All FCF-exposed cell lines showed an increase in the proportion of cells in the G2/M phase (black slices in the pie diagrams on the right) compared to untreated control cells (left pies) pointing towards a blockage of the cells by FCF in the G2/M phase, likely due to inhibition of cytokinesis.

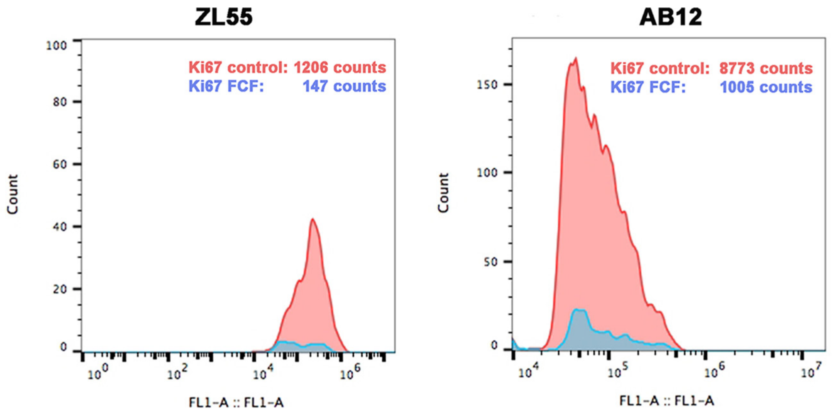

Supplementary Figure 3: Ki67-FACS analysis revealed a strong (> 85%) decrease in proliferating cells in ZL55 (left) and AB12 (right) MM cells exposed to 50 μM FCF for 24 h (blue histograms) compared to untreated control cells (red histograms).

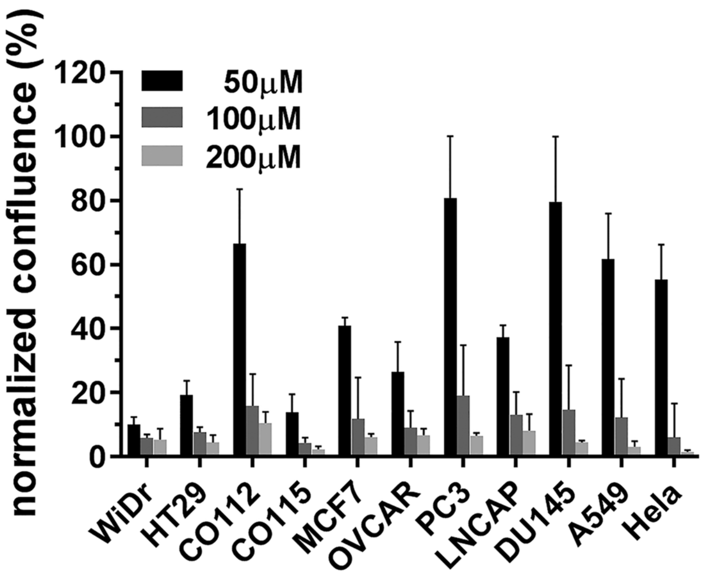

Supplementary Figure 4: A collection of different tumor cell lines from solid tumors of the colon (WiDr, HT29, CO112, CO115), breast (MCF7), ovary (OVCAR), prostate (PC3, LNCAP, DU145), lung (A549) and cervix (HeLa) were exposed to FCF (50, 100, 200 μM). Cell proliferation (determined as % confluence 72 h after start of treatment) was strongly decreased in all FCF-treated cell lines in a concentration-dependent manner. Already at the lowest dose tested (50 μM), a majority of cells lines (6 out of 11) showed a decrease of confluence >50% and all cell lines a decrease of >80% at 100 μM.

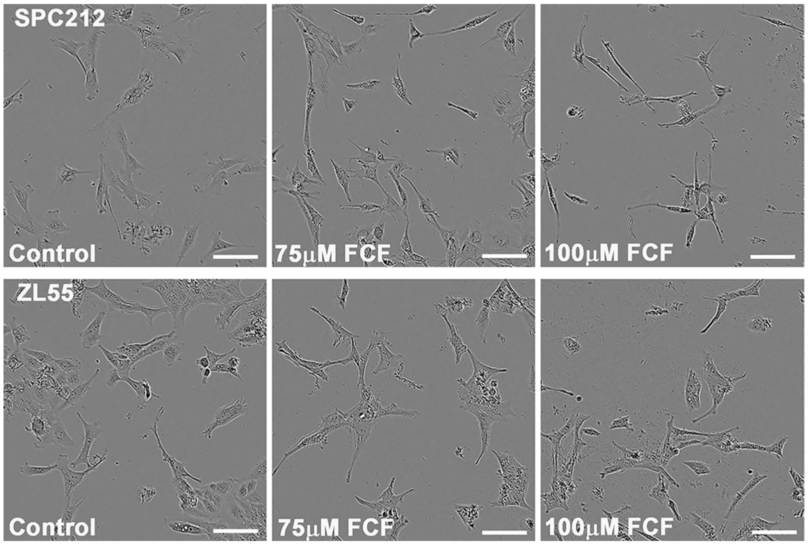

Supplementary Figure 5: SPC212 (upper left) and ZL55 (lower left) control cells were seeded at a lower density to reach approximately similar confluence at 96 h as FCF-treated (75 μM & 100 μM) SPC212 and ZL55 cells. Even when SPC212 (control) cells are seeded at lower density (left), the fraction of elongated (spindloid) cells is rather small compared to a majority of FCF-treated cells showing fibroblast-like (elongated) morphology (middle & right). A similar effect is seen when comparing low-density cultures of ZL55 control cells (left) with FCF-treated (75 μM & 100 μM) ZL55 cells (middle & right). Scale bars: 50 μm.

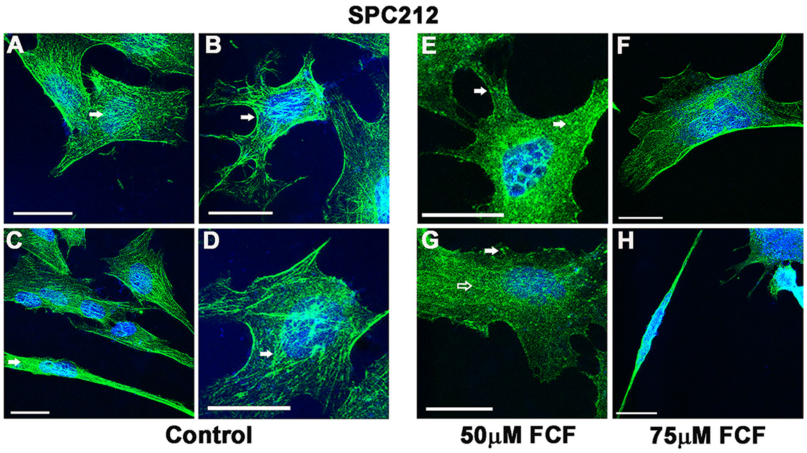

Supplementary Figure 6: Immunofluorescence images of SPC212 cells stained for septin 7 (green) and with Hoechst (blue) to visualize cell nuclei. In control SPC212 cells, septin 7-containing filaments are clearly visible and often resemble actin filament-based stress fibers **(A)** arrow). **(B)** Strong positive staining is also observed along the plasma membrane (arrow). **(C)** In elongated cells (arrow) due to strong staining filaments are less evident. **(D)** Some cells show strong perinuclear staining (arrow). FCF-treated SPC212 cells have different morphologies and distribution of septin 7 fluorescence. **(E)** In the flat epithelioid cell showing signs of blebbing of the nucleus, septin 7 staining shows round puncta in the cytosol and along the plasma membrane (arrows). Moreover, the cytosolic staining is rather homogenous and stronger in the perinuclear region. **(F)** In some cells, septin 7 staining reveals also fiber-like structures and also staining of the plasma membrane region. However, fiber-type structures are less abundant. **(G)** Septin filaments are generally thinner and the organization is less regular (open arrow) compared to control SPC212 cells and again puncta, most often in the cell periphery, are observed (arrow). **(H)** As in the brightfield images (supplementary Figure 5), needle-like cells with strong cytosolic septin 7 staining are present in cultures treated with 75 μM FCF. Scale bar in (A) – (H): 25 μm.

Supplementary Video 1: M1 Control ZL55 cells recorded with the Incucyte Live-Cell Imaging system. Control ZL 55 cells reach near-confluence after the recorded time period (62 h).

See Supplementary Video 1:

Supplementary Video 2: M2 ZL55 cells exposed to 80 µM FCF recorded with the Incucyte Live-Cell Imaging system. FCF-treated ZL55 cells show growth arrest and morphological changes (cell shrinking, membrane blebbing, cell detachment) typical for apoptosis.

See Supplementary Video 2:
